# Carbon assimilation and accumulation of cyanophycin during the development of dormant cells (akinetes) in the cyanobacterium *Aphanizomenon ovalisporum*

**DOI:** 10.3389/fmicb.2015.01067

**Published:** 2015-09-29

**Authors:** Assaf Sukenik, Iris Maldener, Thomas Delhaye, Yehudit Viner-Mozzini, Dotan Sela, Myriam Bormans

**Affiliations:** ^1^The Yigal Allon Kinneret Limnological Laboratory, Israel Oceanographic and Limnological Research, MigdalIsrael; ^2^Faculty of Organismic Interactions, Interfaculty Institute of Microbiology and Infection Medicine Tübingen, University of TübingenTübingen, Germany; ^3^UMS CNRS 3343 Observatoire des Sciences de l’Univers, Université de Rennes 1Rennes, France; ^4^UMR CNRS 6553 Ecosystèmes-Biodiversité-Evolution, Université de Rennes 1Rennes, France

**Keywords:** akinetes, cyanobacteria, dormancy, cyanophycin, NanoSIMS

## Abstract

Akinetes are spore-like non-motile cells that differentiate from vegetative cells of filamentous cyanobacteria from the order Nostocales. They play a key role in the survival and distribution of these species and contribute to their perennial blooms. Here, we demonstrate variations in cellular ultrastructure during akinete formation concomitant with accumulation of cyanophycin; a copolymer of aspartate and arginine that forms storage granules. Cyanophycin accumulation is initiated in vegetative cells few days post-exposure to akinete inducing conditions. This early accumulated cyanophycin pool in vegetative cells disappears as a nearby cell differentiates to an akinete and stores large pool of cyanophycin. During the akinete maturation, the cyanophycin pool is further increased and comprise up to 2% of the akinete volume. The cellular pattern of photosynthetic activity during akinete formation was studied by a nano-metric scale secondary ion mass spectrometry (NanoSIMS) analysis in ^13^C-enriched cultures. Quantitative estimation of carbon assimilation in vegetative cells and akinetes (filament-attached and -free) indicates that vegetative cells maintain their basal activity while differentiating akinetes gradually reduce their activity. Mature-free akinetes practically lost their photosynthetic activity although small fraction of free akinetes were still photosynthetically active. Additional ^13^C pulse-chase experiments indicated rapid carbon turnover during akinete formation and *de novo* synthesis of cyanophycin in vegetative cells 4 days post-induction of akinete differentiation.

## Introduction

Akinetes are resting cells of members of the Nostocales and Stigonematales orders of cyanobacteria. These spore-like, non-motile cells differentiate from vegetative cells and serve a perennial role. Akinetes differ from vegetative cells by cellular structure, composition, and morphology. They are larger than vegetative cells or heterocysts and in some species, can be up to 10-fold larger than vegetative cells. The akinete shape differs among species from sphere to oblate spheroid and their distribution and position within a filament (trichome) is frequently used as a taxonomic feature (Maldener et al., 2014). Akinetes are surrounded by a thickened cell wall and a multilayered extracellular envelope (Nichols and Adams, 1982; Herdman, 1987, 1988). They contain large amounts of reserve materials and essential cellular metabolic pools such as glycogen granules and distinctive cyanophycin globules (CG), but lack gas vesicles (Wildman et al., 1975; Cardemil and Wolk, 1976, 1979; Simon, 1987). The thylakoid system of akinetes is substantially reduced but never completely lost (Miller and Lang, 1968).

Various environmental factors were reported to trigger the differentiation of akinetes; these include light intensity (low or high light), light quality (with preference to green or red light), temperature, and temperature shock as well as temperature fluctuations (Kaplan-Levy et al., 2010). Nutrients are also known to affect the formation of akinetes. Phosphate limitation induced akinete formation (Meeks and Elhai, 2002), but in some cases, phosphate was essential for their full development (Fay, 1988; Moore et al., 2003, 2005; Sukenik et al., 2012). The lack of a clear and common signal that triggers akinete differentiation substantially restricted the research on the developmental process. Recently, we described the formation of akinetes in *Aphanizomenon ovalisporum* in response to potassium ions (K^+^) deficiency (Sukenik et al., 2007, 2013). A burst of akinete formation was observed 3–8 days after K^+^ depletion was imposed, followed by 2–3 weeks of a maturation process.

In our previous studies we demonstrated that akinete differentiation in *A. ovalisporum* (Nostocales) was accompanied by a gradual reduction in the photosynthetic capacity concomitant with variations in the cellular pigmentation, mainly due to loss of phycobilisomes (Sukenik et al., 2007, 2009). We further reported that akinetes contain 15-fold higher chromosome copies than vegetative cells and accumulate ribosomes to a higher level than that found in vegetative cells (Sukenik et al., 2012). This massive accumulation of nucleic acids in akinetes was probably supported by phosphate supplied from inorganic polyphosphate bodies that were abundantly present in vegetative cells, but notably absent from mature akinetes.

Here, we report on the accumulation of cyanophycin during akinete formation. This nitrogenous reserve material is a copolymer of aspartate and arginine that forms storage granules. Our results indicate that cyanophycin accumulation is enhanced in vegetative cells few days post-exposure to akinete inducing conditions. This early accumulated cyanophycin pool in the vegetative cells is diminished as nearby cells differentiate to akinetes. During the akinete maturation, their cyanophycin storage pool is further increased.

## Materials and Methods

### Culture Conditions and Formation of Akinetes

*Aphanizomenon ovalisporum* (Forti) strain ILC-164 (Banker et al., 1997) was maintained in batch cultures in BG11 medium (Stanier et al., 1971). Cultures were grown at 25°C with air bubbling (2 L min^-1^) under continuous illumination of 45 μmol quanta m^-2^ s^-1^. For the induction of akinete formation, trichomes from 10-day-old exponential cultures were harvested by centrifugation and transferred to an akinete-inducing medium (a BG11 medium deprived of potassium ion by substituting the K_2_HPO_4_ component with Na_2_HPO_4_) as previously described (Sukenik et al., 2007). Akinete-induced cultures were maintained for 2–3 weeks at 25°C under continuous illumination of 45 μmol quanta m^-2^ s^-1^ during which akinetes were formed. Two categories of akinetes were defined, filament-attached akinetes and mature-free akinetes. The trichome vegetative cells (named vegetative cells hereafter) had an average wet weight biomass of 0.05 ng (w.w.), whereas the size of filament-attached akinetes increased as their differentiation proceeded and they exceeded a biomass of 0.4 ng (w.w.; Sukenik et al., 2013). During their differentiation, akinetes were characterized by a thickening of their cell wall and by the accumulated storage globules. The average biomass of young akinetes was 0.1 ng (w.w.). At later stages of their development, akinetes reached an average biomass of 0.4 ng (w.w.) and eventually detached from the trichome. These free akinetes were defined as mature (Kaplan-Levy et al., 2015).

### Sample Preparation and Transmission Electron Microscopy (TEM) Observation

Samples for transmission electron microscopy were prepared as described previously (Fiedler et al., 1998). In brief, fixation and post-fixation were performed using glutaraldehyde and potassium permanganate. Fixed samples were embedded in EPON and ultra-thin sections (60–80 nm) were stained with uranyl acetate and lead citrate. The sections were examined with a Philips Tecnai electron microscope at 80 kV.

### Staining of Cellular Cyanophycin Pools

For visualization of CG in the microscope, we used a novel staining procedure based on the Sakaguchi reaction (Messineo, 1966) as modified by Watzer et al. (submitted). CG were stained dark purple and the color was stable for at least several hours when kept in dark. Stained samples were examined under Zeiss Axio Observer Z1 inverted microscope using 63X objective (Zeiss Pla-apochromat 63X/1.40 oil DIC M27) and bright field optics. A series of images of stained filaments and akinetes were recorded using Zeiss AxioCam camera for subsequent processing and image analysis.

### Image Analysis

Microscopic micrographs were analyzed using basic functions for image adjustment and processing of Image J, a public domain Java image-processing program (http://imagej.nih.gov/ij/). For cyanophycin quantification, the number of globules per cell and the area of each globule were determined by using the Analyze Particles procedure that calculate area and pixel value statistics of user-defined region of interest (ROI). A nominal radius for the aerial projection of cells (vegetative and akinetes) and CG was calculated, assuming a circular shape. The calculated radius was used to estimate the corresponding volume assuming spherical shape for both cells and CG. The total volume of cellular cyanophycin pool was calculated as a product of globules per cell and the average volume of a cyanophycin globule.

### Culture Labeling with Stable Isotopes and Analysis by Nanometric Scale Secondary Ion Mass Spectrometry (NanoSIMS)

For assessment of photosynthetic carbon assimilation during the induction of akinete formation, a 500-mL *A. ovalisporum* culture maintained in akinete-inducing conditions was sampled at time zero and 8 and 12 days post-induction, indicating formation and maturation processes, respectively. Subsamples of 75 mL were washed and re-suspended in a fresh akinete inducing medium and further incubated in 150 mL Erlenmeyer flask under the conditions described above after the addition of 0.1 mL NaH^13^CO_3_ (ca.100 atom percentage ^13^C, 0.04 M, final ^13^C enrichment 1.9 atom percentage DIC; Cambridge Isotope Laboratories). These cultures were sampled at three time points (0, 3, and 6 h) to follow carbon assimilation rate and ^13^C enrichment. At each time, 20 mL sample was withdrawal from the culture, washed in phosphate buffered saline –PBS (50 mM Phosphate buffer pH 7.8 in 0.9% NaCl) and fixed with 2% paraformaldehyde in PBS for 1 h. Fixed samples were washed re-suspended in PBS and kept at 4°C until filtration onto RTTP filters (1.2 μm pore size, 25 mm diameter, Merck Millipore). RTTP filters were pre-coated with a gold thin layer to enhance the conductivity. The filters were kept at -80°C until freeze-drying for 24 h. Filters were cut at a diameter of 5 mm and mounted on a sample holder. Samples were analyzed by a NanoSIMS 50L (Cameca, Gennevilliers, France) which allows analysis of elemental and isotopic composition of solid samples at a sub-micrometer (down to 50 nm). Spatial resolution of 50 nm scale is achieved using a focus ion beam that hits and erodes the surface of the sample. The high mass resolution (M/DM > 5000) allows the separation of ions of very close masses. The analyses were done using the Cs^+^ primary ion source to detect negative ions. For individually observed frame, total ion content (TIC) image and secondary ion images for ^12^C, ^13^C, and ^12^C^14^N were recorded. The data processing and image analyses were performed using the ImageJ software (Schneider et al., 2012).

The fate of carbon assimilated during akinete differentiation and its potential contribution to akinetes’ storage pools was further studied in a pulse chase experiment where ^13^C bicarbonate was provided to akinete-induced cultures for the first 2 days of induction and chased with ^12^C bicarbonate for additional 20 days. Subsamples were collected from various stages of this experiment fixed and sectioned for further analysis by NanoSIMS. Sample preparation included fixation in 2.5% glutaraldehyde for 1 h, washing with 0.1 M sodium cacodylate buffer (pH 7.4) and embedding in 1% Seakem agarose in 0.1 M sodium cacodylate buffer (pH 7.4). The samples were dehydrated and further embedded in an epoxy resin. A Leica Ultracut (UCT) ultramicrotome was used for resin sectioning to provide 500 nm slices. These sections were analyzed by the Cameca NanoSIMS 50L ion microprobe to detect sub-micrometer localization of isotopically enriched structures.

### Nano-scale SIMS Data Processing

Ion species ^12^C- and ^13^C- were used to detect carbon associated with biomass and ^13^C/^12^C ratio was used to assess carbon uptake and assimilation (enrichment over the background), during the labeling period. Isotopic ratio color images were generated by using OpenMIMS, an ImageJ plugin developed by Claude Lechene’s Laboratory (Poczatek et al., 2009). OpenMIMS allows the visualization of the ratio value in a Hue–Saturation–Intensity (HSI) image. Additional ImageJ plug-ins were used to compute the average ^13^C/^12^C ratios in the cells (Chew et al., 2014).

## Results

### Ultrastructural Variations during Akinete Differentiation and Maturation

**FIGURE 1:**
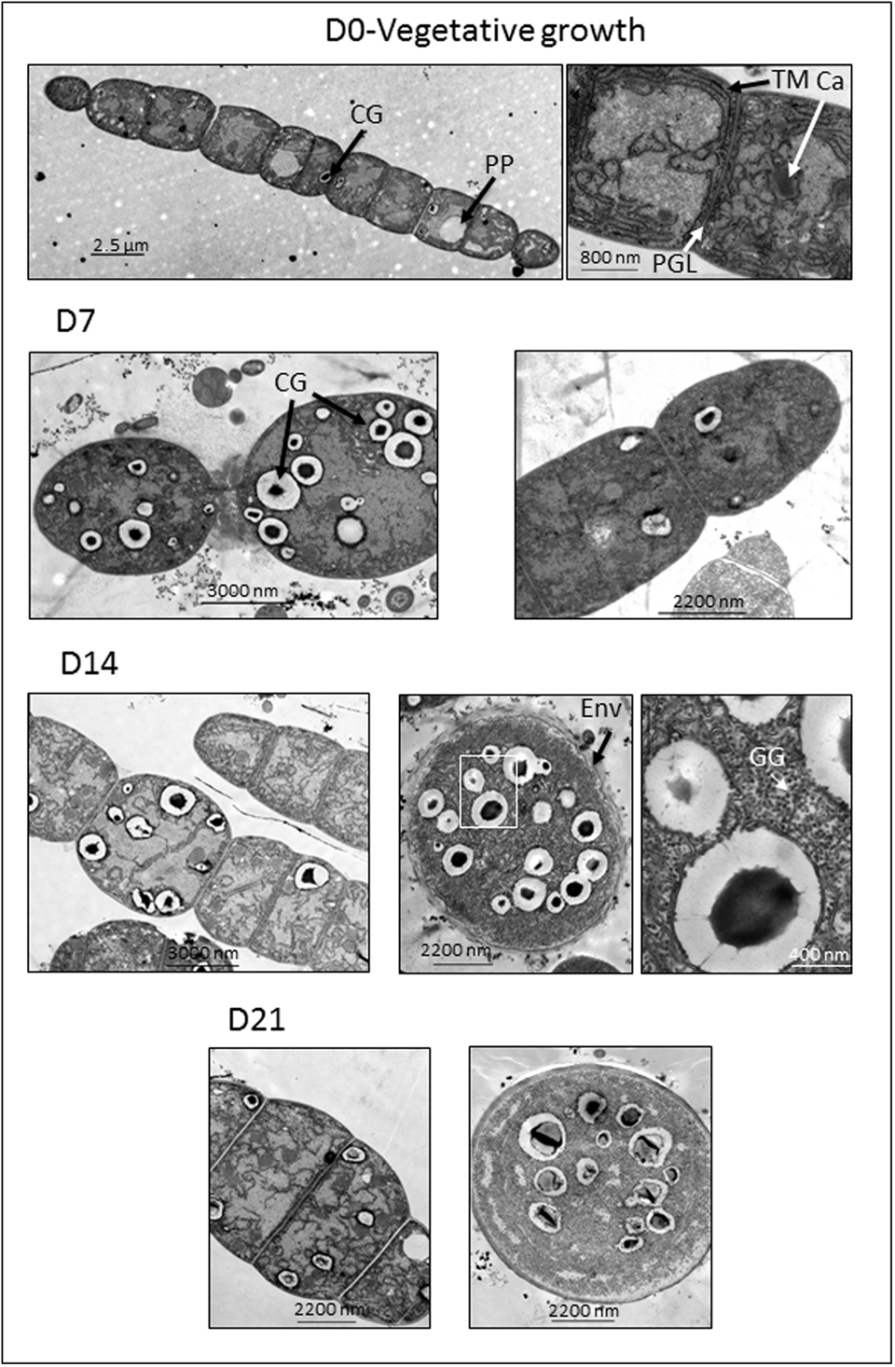
**Electron micrographs of ultra-thin sections of filaments and akinetes of *Aphanizomenon ovalisporum* captured at different time points (D0, day 0; D7, day 7; D14, day 14; and D21, day 21) after akinete-induction (transfer to K^+^-depleted medium).** Ca, carboxysome; CG, cyanophycin globule; GG, glycogen globule; Env, envelope; PP, polyphosphate body; TM, thylakoid membrane. The right electron micrograph of D14 lane is the magnified part indicated by a white frame of the middle micrograph. A scale bar is presented for each micrograph.

**FIGURE 2:**
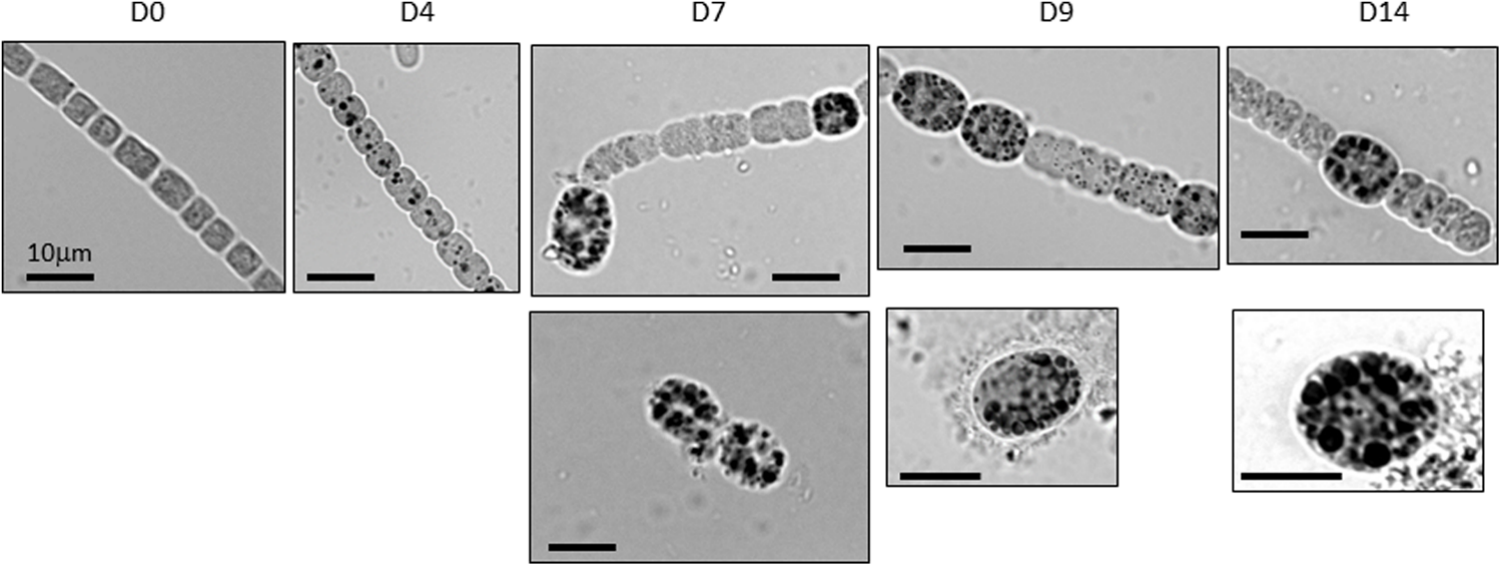
**Accumulation of cyanophycin globules (CG) in vegetative cells and akinetes in *A. ovalisporum* incubated under akinete-inducing conditions.** Samples collected at different times during the induction (D0, D4, D7, D9, and D14, represent sampling days) were stained by Sakaguchi reaction and CG became dark visible. Filament-attached akinetes of different developmental stages are visible in D7, D9 and D14 (upper row) together with free akinetes (lower row). Scale bars represent 10 μm.

**FIGURE 3:**
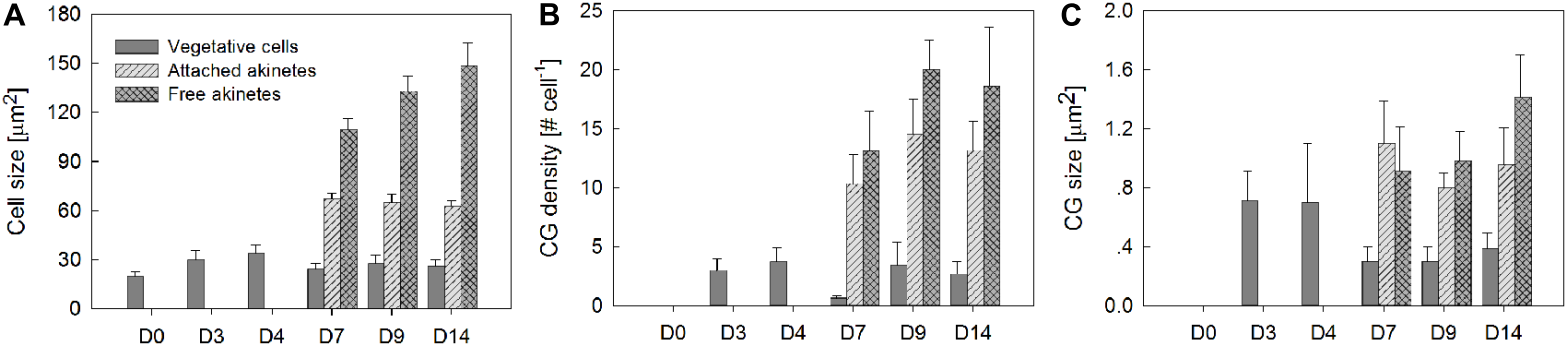
**Quantitative data of cell size **(A)**, cellular abundance of cyanophycin globule **(B)** and the size of cyanophycin globule **(C)** in vegetative cells and akinetes (filament-attached and -free) in *A. ovalisporum* incubated under akinete-inducing conditions.** Samples collected at different times during the induction (D0, D4, D7, D9, and D14, represent sampling days) were stained by Sakaguchi reaction and CG became dark visible (as presented in **Figure 2**). Data was collected by analyzing microscopic images using ImageJ, image-processing program. Bars present average and standard deviation. For each time point and cell type at least 40 individual objects were analyzed.

Vegetative cells of *A. ovalisporum*, prior to implementation of akinete inducing conditions (K^+^ deficiency), showed typical cellular ultrastructure of filamentous cyanobacteria (Maldener et al., 2014) (**Figure 1**, D0-vegetative growth). This includes advanced structure of thylakoid membranes in the periphery of the cell and surrounding the carboxysomes, where ribulose bisphosphate carboxylase/oxygenase (RUBISCO) localizes. As in other Nostocales (Flores et al., 2006), a continuous outer membrane of the cell wall encompasses all cells of the filament. A peptidoglycan layer is visible in the septum between neighboring cells. Sporadic CG, polyphosphate bodies and small dark globules, presumably polyglucose bodies (glycogen globules), are visible in some sections (**Figure 1**, D0-vegetative growth). Under akinete-inducing conditions, the well-organized thylakoid membranes are distorted and the abundance of CG increases. The size of vegetative cells that initiated their differentiation to akinetes increases and high number of CG of various sizes appears (**Figure 1**, D7 left image; see also **Figures 2** and **3**). As the akinete differentiation progresses, the cell size further increases and less thylakoids are observed. Fourteen days after akinete induction, a multilayered envelope structure, surrounding the developing akinete becomes visible. Free akinetes contain large numbers of CG of different sizes and are loaded with glycogen globules, which fill the cytoplasm (**Figure 1**, D14 right panels and D21 right panel). In general, the development of akinetes did not occur synchronously as evident in the filament attached CG enriched young akinete shown in **Figure 1**, D14 left panel. However, compared to non-induced cultures (**Figure 1**, D0) vegetative cells of old akinete-induced cultures show cellular modifications such as reduction of thylakoid membranes, concomitant with low amounts of reserve material relative to mature akinetes (**Figure 1**, D21).

### Accumulation of Storage Polymers in Akinetes

Accumulation of cyanophycin during akinete differentiation was further studied and confirmed using specific histochemical procedures followed by light microscopic examination and image analysis. Representative images show accumulation of CG in vegetative cells of akinete-induced cultures (4 days post-induction), prior to any visual akinete differentiation (**Figure 2**, D4). Later on, as a differentiating akinetes extended their size, they accumulated CG (**Figure 2**, D7 and D9) and CG pools in the adjacent vegetative cells was reduced (**Figure 2**, D7) and eventually the CG pool in vegetative cells between two developing akinetes diminished (**Figure 2**, D14). As akinetes matured and detached from the filament, they further accumulated CG of variable sizes and quantities, as also observed in electron micrographs (**Figure 1**, D21). Images collected during the akinete differentiation process were quantitatively analyzed for cell size (expressed in aerial units extracted from 2D images) and for CG cellular abundance and size (**Figure 3**). Upon exposure to akinete-inducing conditions, the size of vegetative cells initially increased but as akinetes formed, vegetative cells shrank back to their original size (**Figure 3A**). Filament-attached and -free akinetes appeared 7 days (D7) post-induction and their size was 3–5 times larger than vegetative cells. CG (ca. 4 per cell) were observed in vegetative cells 3 days post-induction and their density substantially reduced (less than 1 per cell) as akinetes formed on day 7, and slightly increased later on (**Figure 3B**). The average size of the CG accumulated in vegetative cells during the early stage of akinete induction decreased significantly, as akinetes formed (from ca. 7 to less than 4 μm^2^, **Figure 3C**). Filament-attached akinete contained between 5- and 10-fold more CG than the vegetative cells whereas free akinetes contained more CG than attached akinetes. The average size of the CG accumulated in akinetes was 2–3 times larger than in vegetative cells. The average size of the CG accumulated in free akinetes 14 days post-induction (D14) was significantly larger (*p* < 0.05, *n* = 15) than in attached akinetes (**Figures 3B,C**).

### Carbon Assimilation during Akinete Differentiation

**FIGURE 4:**
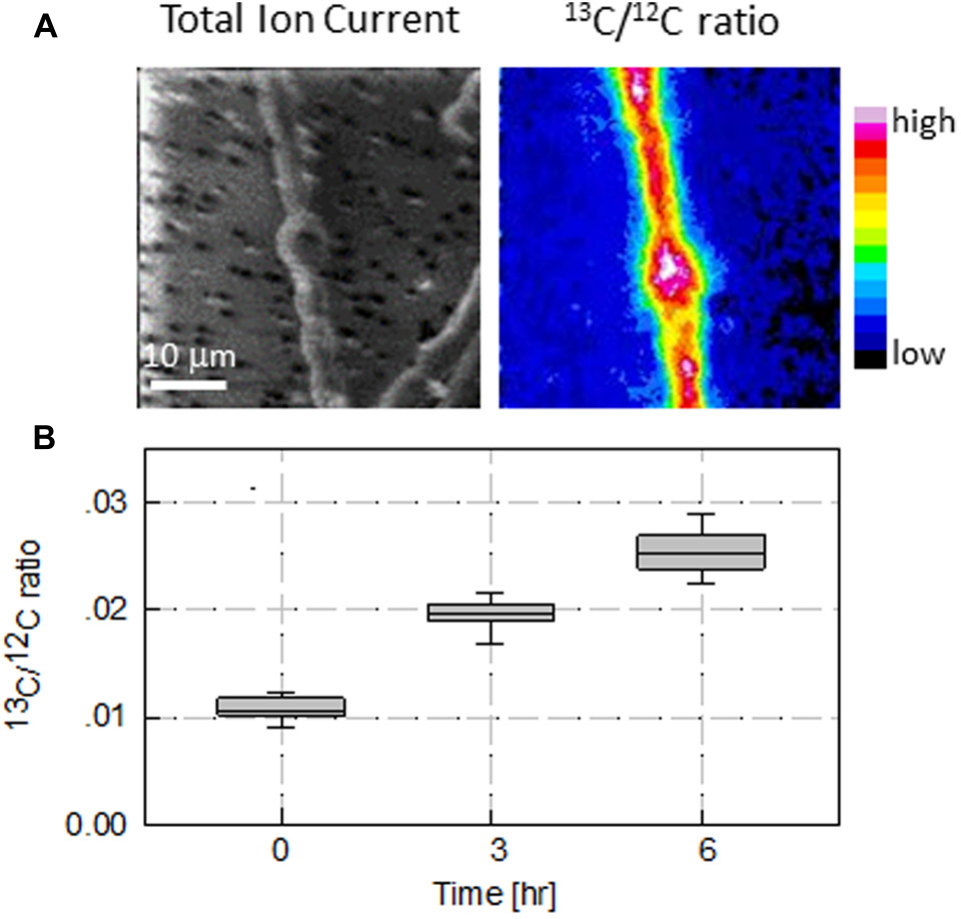
**Carbon assimilation in vegetative cells and akinetes in akinete induced culture as measured by nanometric scale secondary ion mass spectrometry (NanoSIMS). (A)** Ion micrographs of vegetative cells and a young akinete and **(B)** quantitative estimation of carbon assimilation in vegetative cells incubated with ${\text{H}}^{\text{13}}{\text{CO}}_{\text{3}}^{-}$ for 6 h. Images in **(A)** are of total ion count (TIC) – left panel, and calculated ^13^C/^12^C ratio based on NanoSIMS analysis (color scale ranged from 0.0115 to 0.0275) – right panel. The summarized data in the box plot **(B)** indicates time dependent increase in ^13^C/^12^C ratio in photosynthetically active vegetative cells. The lower boundary of the box indicates the 25th percentile, a solid line within the boundary marks the average and the upper boundary of the box indicates the 75th percentile. Whiskers above and below the box indicate the 95th and 5th percentiles.

Photosynthetic carbon assimilation by vegetative cells and differentiating akinetes was evaluated using nanometric scale secondary ion mass spectrometry (NanoSIMS) to scan samples incubated with ^13^C bicarbonate. Typical NanoSIMS images of a short filament with a single akinete (representing 8 days old akinete-induced culture) are presented in **Figure 4A**. The total ion current image shows a filament with an attached akinete. The image for ^13^C/^12^C ratio represents values calculated from ^13^C and ^12^C ions images acquired after 6 h incubation with the isotopic tracer. This ^13^C/^12^C ratio image indicates carbon assimilation in both vegetative cells and akinetes at the specific stage of the akinete-induced culture (8 days). Acquiring ^13^C and ^12^C ion images from filaments of 8 days old akinete-induced culture after 3 or 6 h incubation with ^13^C, demonstrate photosynthetic carbon assimilation as the ^13^C/^12^C ratio in vegetative cells increased linearly from a background value of 0.012 (representing natural abundance of ^13^C) at time zero to 0.027 after 6 h (**Figure 4B**).

**FIGURE 5:**
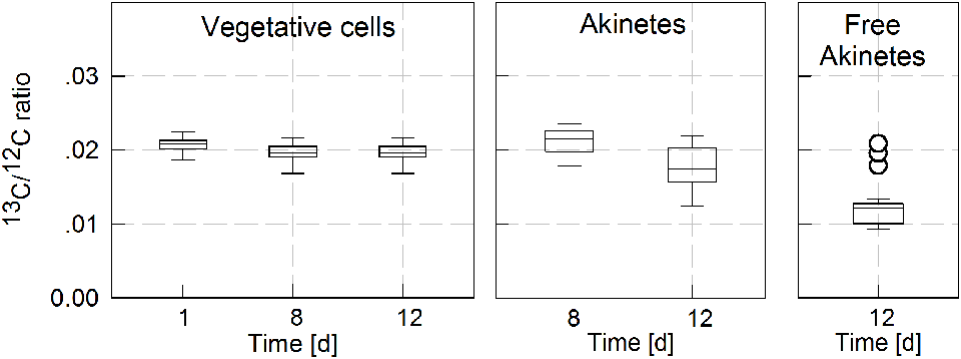
**Quantitative estimation of carbon assimilation in vegetative cells and akinetes (filament-attached and -free).** *A. ovalisporum* culture was exposed to akinete inducing conditions for 12 days. Subsamples were collected at time intervals (1, 8, and 12 days) and incubated with ${\text{H}}^{\text{13}}{\text{CO}}_{\text{3}}^{-}$ for 3 h. Carbon assimilation to vegetative cells and akinetes is indicated by ^13^C/^12^C ratio calculated from NanoSIMS analysis. Data presented as box plot is grouped for vegetative cells, filament-attached akinete and -free akinetes. The box plot present the all collected data for each case (between 10 and 40 individual objects were analyzed). The lower boundary of the box indicates the 25th percentile, a solid line within the boundary marks the average and the upper boundary of the box indicates the 75th percentile. Whiskers above and below the box indicate the 95th and 5th percentiles. In the free akinetes panel, the open circle symbols represent individual akinetes with exceptionally high ^13^C/^12^C ratio.

The NanoSIMS procedure was further employed to evaluate photosynthetic carbon assimilation in vegetative cells and akinetes during akinete formation in *A. ovalisporum* cultures. Images of ^13^C/^12^C ratio were produced for vegetative cells and akinetes. The accumulated data presented in **Figure 5** indicate that vegetative cells maintained their photosynthetic capacity for a relatively long period (at least 12 days) during which akinetes were formed. A substantial enrichment in ^13^C was observed in vegetative cells and the ^13^C/^12^C ratio reached value of 0.02 (after 3 h incubation) with no significant variations during the entire induction period. Filament-attached akinetes in 8 days old induced culture, maintained photosynthetic activity at a similar (or even slightly higher) rate as vegetative cells. In an older culture, the photosynthetic capacity of attached akinetes slightly declined and the average ^13^C/^12^C ratio was 0.0175, although relatively high variability was recorded. Free akinetes were observed in 12 days old induced culture and their recorded ^13^C/^12^C ratio was variable. Most of the analyzed free akinetes maintained only residual photosynthetic activity with an average ^13^C/^12^C ratio of 0.013. However, ^13^C/^12^C ratios as high as 0.018 (represented by open circles in free akinetes panel of **Figure 5**) were recorded in few free akinetes.

**FIGURE 6:**
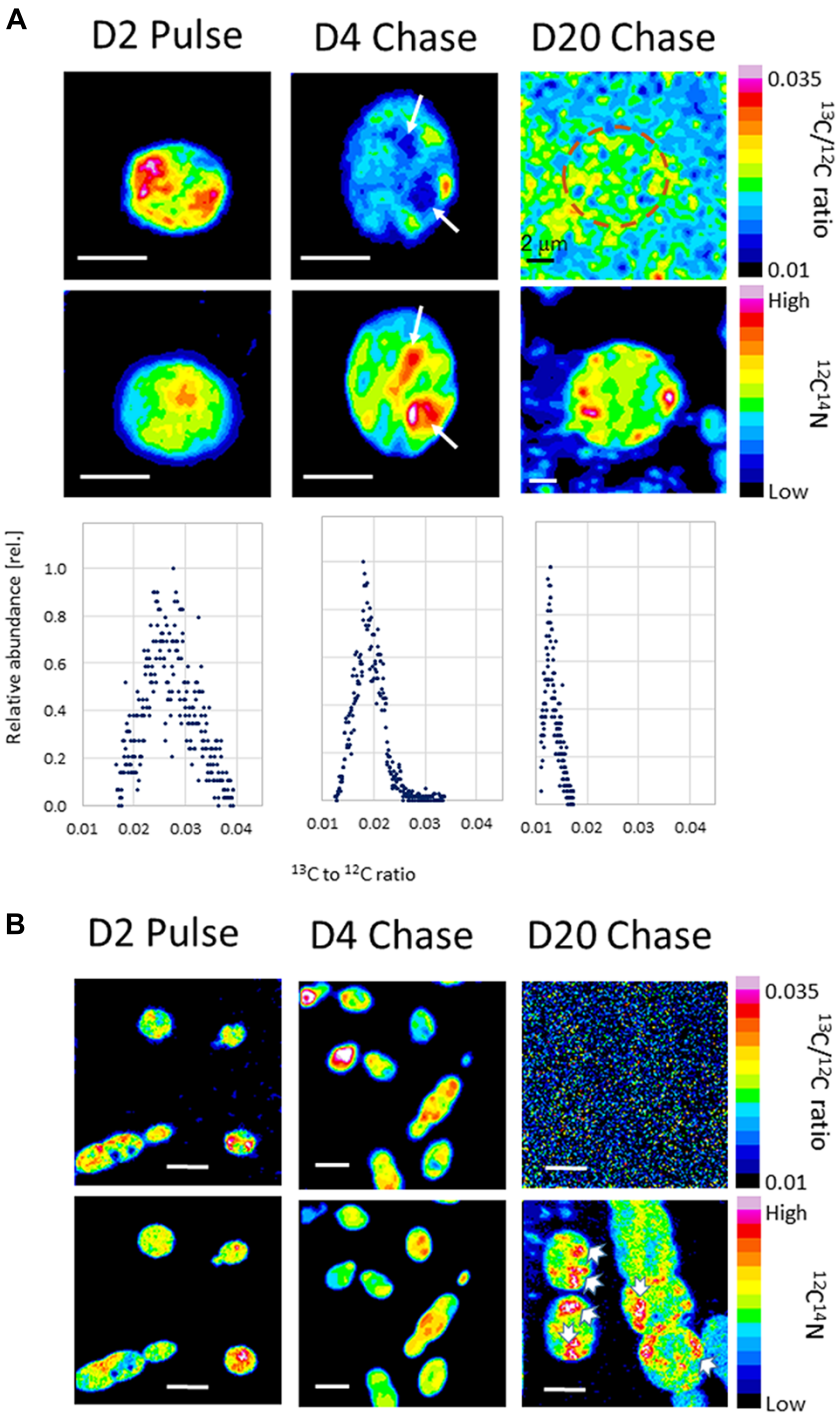
**Secondary ion images of **(A)** vegetative cells (D2 Pulse and D4 Chase) and of an akinete (D20 Chase) of *A. ovalisporum* and **(B)** corresponding filaments.** Images are based on NanoSIMS analysis. ^13^C bicarbonate was provided to akinete-induced cultures for the first 2 days and chased with ^12^C bicarbonate for additional 18 days during which akinete differentiation occurred. Images in **(A)** represent transverse section of vegetative cells or an akinete. Note size differences between vegetative cells and akinetes. **(A)** The upper row images represent ^13^C to ^12^C ratio. Note the white arrows in D4 Chase image indicating new synthesis of presumable cyanophycin. The D20 Chase image indicates reduced ^13^C signal (^13^C to ^12^C ratio near the background level) which masks the location of an akinete (presented by a dotted circle which was allocated based on the overlay with the corresponding ^12^C^14^N ion image). The lower row images show the localization of N-enriched compounds based on the ^12^C^14^N ion. Bar scales = 2 μm. Color scales for images is provided. The graphs at the lower row show the relative distribution of ^13^C to ^12^C ratio of more than 2000 pixels composed each cellular image, indicating ^13^C dilution during the chase period. **(B)** The upper row images represent ^13^C to ^12^C ratio in several filaments. Note the D20 Chase image with substantially reduced ^13^C signal (^13^C-to-^12^C ratio near the background level) which masks the location of the filaments. The lower row images show the localization of N-enriched compounds in the same filaments based on the ^12^C^14^N ion. Note the localization of high ^12^C^14^N ion signal in D20 Chase image, presumably presented cyanophycin (indicated by white arrows) in free akinetes and in attached akinetes. Bar scales = 4 μm. Color scales for images is provided.

The fate of the assimilated carbon was further studied in a pulse chase experiment using akinete-induced cultures. After 2 days of incubation with ^13^C bicarbonate, the ^13^C/^12^C ratios in cells increased from a background value of 0.012 to an average value of 0.028. The image of ^13^C/^12^C ratio presented in **Figure 6A** (D2 pulse) for an individual vegetative cell shows uneven distribution and local amplification of ^13^C/^12^C isotopic ratio with maximal value of 0.035, almost three times higher than the background. The ^12^C^14^N ion image of the same cell indicate different localization pattern as compared to the ^13^C/^12^C isotopic ratio. This different cellular localization is further noticed in other vegetative cells and indicate variability among cells on the same filament (**Figure 6B**, D2 Pulse). Chasing the ^13^C isotope for additional 2 days imposed substantial decrease in the cellular ^13^C/^12^C isotopic ratio that decreased to an average value of 0.019 with high values localized in confined areas (**Figure 6A**, D4 chase). The low ^13^C/^12^C isotopic ratio zone indicated by white arrows (**Figure 6A**, D4 chase) present *de novo* synthesis that could be attributed to a newly accumulation of CG. The ^12^C^14^N ion image of the same cell indicates the presence of N-enriched compounds at the same cellular region, which presumably were synthesized during the 2 days chase period. Similar trend of accumulation of N-enriched compounds poorly labeled with ^13^C was evident in other single cells and short filaments presented in **Figure 6B** (D4 chase). Samples collected after longer chase period (D20) contained free akinetes as the one presented in **Figure 6A** (D20, note the large dimension, a diameter of 10 μm, as compared to 3 μm of vegetative cells). The long chasing period left the cells (both akinetes and vegetative) substantially depleted of the heavy C isotope, but the ^12^C^14^N ion image of the akinete indicates the presence of N-enriched compounds at well-defined regions (**Figure 6A**, D20). Similar region of N-enriched compounds were located in both, free and filament attached akinetes (**Figure 6B**, D20).

## Discussion

In prokaryotes, cell differentiation evolved as survival strategy and adaptation to changing environments. Filamentous cyanobacteria can undergo a variety of cellular differentiation processes forming dormant akinetes, nitrogen fixing heterocysts and motile hormogonia (Maldener et al., 2014). Although akinetes of different Nostocales species differ in their morphological characteristics, they still share many common features but clearly differ from vegetative cells. Akinetes are larger than vegetative cells or heterocysts and in some species, can be up to 10-fold larger than vegetative cells. Here, we show that the cell size of mature akinetes from *A. ovalisporum* can be up to five times bigger than vegetative cells. Thickened cell wall and a multilayered extracellular envelope were described for akinetes from different strains (Nichols and Adams, 1982; Herdman, 1987, 1988) similar to the multilayered envelope presented in akinetes of *A. ovalisporum* (**Figure 1**). Akinetes of *A. ovalisporum* contain distinctive granules of cyanophycin and glycogen, but lack gas vesicles as reported for many other species of Nostocales (Wildman et al., 1975; Cardemil and Wolk, 1976, 1979; Simon, 1987). Other distinct cellular features of *A. ovalisporum* are reduced thylakoid system, substantial reduction in the phycobilisome pool and genome multiplication to high level of polyploidy (Sukenik et al., 2007, 2012).

While various environmental signals were suggested to trigger akinete differentiation (Kaplan-Levy et al., 2010), the precise cascade of cellular signals that governs the differentiation process and pattern formation is yet to be identified. In an early study, we reported that deprivation of potassium ion (K^+^) triggers akinete development in the cyanobacterium *A. ovalisporum*. Akinete formation initiated 3–7 days after an induction by K^+^ depletion, followed by 2–3 weeks of a maturation process. In a recent study, we negated the possible involvement of reactive oxygen species (ROS) in the regulation of akinete differentiation process (Kaplan-Levy et al., 2015). Although the internal signal, that triggers the differentiation of a given vegetative cell to akinete has yet to be identified, the observations that this is a non-synchronized process and that only few vegetative cells undergo this differentiation route, suggest that cell–cell communication is involved in the decision (Maldener et al., 2014).

While biochemical and metabolic features of vegetative cells versus mature akinetes are well documented, gradual modification of cellular pools such as cyanophycin is at the center of our current study. Under akinete induction conditions, CG accumulated in vegetative cells, 4 days post-induction. These C/N storage pools diminished as one of the adjacent vegetative cells initiated its differentiation to an akinete, concomitant with substantial increase in cyanophycin granules inside the developing akinete (**Figures 1–3**). For example the average calculated CG pool of a vegetative cell, 4 days post-induction was about 2 μm^3^ (representing 1.5 % of the total cell volume). Furthermore, results of the pulse chase experiment suggest that accumulation of CG in vegetative cells, 4 days post-induction, was built mainly from *de novo* photosynthetic assimilated carbon (**Figure 6**). Three days later (D7), this vegetative cell pool was reduced (the calculated cellular volume of CG was only 0.08 μm^3^) as an adjacent cell initiated its differentiation to an akinete with an average calculated CG pool of about 9 μm^3^ (representing 2% of the total cell volume). Interestingly, the volume of an averaged attached akinete increased more than six times relative to the volume of a vegetative cell at D0. At this developmental stage, one may calculate that the CG pool of 4–5 vegetative cells was “translocated” into the differentiating akinete. As akinetes further matured CG accumulation in akinetes continues and when detached from the filament, additional CG were accumulated to an averaged CG pool of more than 25 μm^3^ in free akinetes (but still representing 2% of the akinete volume). Due to rapid dilution of the ^13^C signal in vegetative cells and akinetes, CG “translocation” from vegetative cells to akinetes could not be confirmed.

As filament-attached akinetes maintain their photosynthetic activity, carbon allocation for *de novo* synthesis of CG cannot be excluded. Finzi-Hart et al. (2009) performed correlated transmission electron microscopy (TEM) and NanoSIMS analysis on trichome thin-sections of *Trichodesmium* and observed transient inclusion of ^15^N and ^13^C into discrete subcellular bodies identified as cyanophycin granules. They speculated that *Trichodesmium* uses these dynamic storage bodies to uncouple CO_2_ and N_2_ fixation from overall growth dynamics. Observation and incubation experiments with cultures and field samples using NanoSIMS approach revealed the presence of abundant cyanophycin granules in photosynthetically active cells of non-heterocystous filamentous cyanobacteria from coniform mats (Liang et al., 2014).

The cellular pattern of photosynthetic activity during akinete formation documented in this study by NanoSIMS analysis indicated that vegetative cells maintained their basal activity while differentiating akinetes gradually reduced their activity. Mature-free akinetes practically lost their photosynthetic activity although small fraction of free akinetes were still photosynthetically active (**Figure 6**). Similar results were previously reported based on measurements of cellular activity using Microscope pulse amplitude modulated (PAM). In that study we measured changes in the photosynthetic activities of individual vegetative cells and akinetes in trichomes of *A. ovalisporum* during akinete formation and showed that mature isolated akinetes retained only residual photosynthetic capacity as the photosynthetic machinery was modified. In matured akinetes of *A. ovalisporum* the phycobilisome antenna was reduced in size and apparently detached from the reaction centers (Sukenik et al., 2007).

Accumulation of CG in akinetes was reported for many Nostocales species (Sarma and Khattar, 1993; Sarma et al., 2004). For example in *Nostoc* PCC 7524, the mean cellular content of cyanophycin was eightfold higher than in vegetative cells (Sutherland et al., 1979). Accumulation of cyanophycin was not specific for akinete development; vegetative cells also accumulated cyanophycin when entering the stationary phase (Herdman, 1987). In *A. ovalisporum*, accumulation of CG and akinete differentiation co-occurred under the right environmental conditions (Sukenik et al., 2013). However, under potassium deficiency and temperatures higher than 28°C, neither akinetes differentiation nor CG accumulation were observed (unpublished results). Incubation of *Anabaena cylindrica* with the arginine analog, canavanine (Nichols et al., 1980; Nichols and Adams, 1982) and mutation of the arginine biosynthesis gene, *argL*, in *Nostoc ellipsosporum* (Leganés et al., 1998), resulted in the production of akinetes lacking cyanophycin, but being unable to germinate. Thus, it was suggested that cyanophycin accumulation is not essential for the formation of akinetes, but for its germination, leaving the role of cyanophycin in akinetes during formation and dormancy unresolved.

Cyanophycin, a water-insoluble reserve polymer is a product of non-ribosomal peptide synthesis by cyanophycin synthase, coded by *cphA* (Ziegler et al., 2001). Synthesis of cyanophycin requires ATP, the constituent amino acids aspartic acid and arginine, and a primer such as β-Asp-Arg, or (β-Asp-Arg)_3_ (Berg et al., 2000). The degree of polymerization of the formed cyanophycin is quite variable with a mass range between 25 and 100 kDa (Simon, 1971). Cyanophycin is degraded by the enzyme cyanophynase coded by *cphB*. The cyanophynase enzyme, appears to be specific for cyanophycin, hydrolyses the polymer to a dipeptide consisting of aspartic acid and arginine (Richter et al., 1999). Early studies suggested that cyanophycin serves as a dynamic reservoir, which separates the environmental supply of fixed nitrogen from the metabolic demands of the cells (Mackerras et al., 1990; Kolodny et al., 2006). In single cell cyanobacteria, the dynamics of cyanophycin synthesis and degradation was attributed to environmental conditions but in filamentous cyanobacteria (i.e., Nostocales) cyanophycin may be accumulated in a given cell but serve as an N reservoir for other cells on the same filament. For example, the heterocyst pole (known as polar plug) consisting of cyanophycin (Lang and Fay, 1971; Lang et al., 1972; Ziegler et al., 2001; Herrero and Burnat, 2014). Sherman et al. (2000) suggested that the cyanophycin-containing polar plug is a key intermediate in the storage of fixed nitrogen in the heterocyst, serving as a dynamic reservoir of fixed nitrogen within heterocysts (Carr, 1988). The relevance of the accumulation of cyanophycin at the polar region is yet unclear (Ziegler et al., 2001). Recent studies showed that intercellular exchange of small hydrophilic molecules is reduced when cyanophycin was present in the polar plug of heterocyst of *Anabaena variabilis* ATCC 29413 (Mullineaux et al., 2008) but that was not the case in *Cylindrospermopsis raciborskii* (Plominsky et al., 2015).

“Translocation” of cyanophycin pool from vegetative cells to a differentiating akinete requires hydrolysis of the polymer by cyanophynase and transfer of the products, presumably β-Asp-Arg, to the differentiating akinete where it is reused by cyanophycin synthase to rebuild the polymer. This pathway requires coordinated expression of CphA and CphB proteins to variable levels and at different timing in vegetative cells and in differentiating akinetes during the differentiation process. Variation in the transcription level of these genes along the filament and during akinete differentiation need to be deciphered and the “translocation” hypothesis should be verified by demonstrating intercellular transfer of CG building blocks and substrates. Furthermore, it would be interesting to know how this major reserve pool is maintained during dormancy and how it serves the germination process.

## Conflict of Interest Statement

The authors declare that the research was conducted in the absence of any commercial or financial relationships that could be construed as a potential conflict of interest.
